# Polymorphisms cMyc-N11S and p27-V109G and breast cancer risk and prognosis

**DOI:** 10.1186/1471-2407-7-99

**Published:** 2007-06-14

**Authors:** Jane C Figueiredo, Julia A Knight, Stewart Cho, Sevtap Savas, U Venus Onay, Laurent Briollais, Pamela J Goodwin, John R McLaughlin, Irene L Andrulis, Hilmi Ozcelik

**Affiliations:** 1 Samuel Lunenfeld Research Institute, Mount Sinai Hospital, Toronto, Ontario, Canada; 2 Department of Pathology and Laboratory Medicine, Mount Sinai Hospital, Toronto, Ontario, Canada; 3 Division of Preventive Oncology, Cancer Care Ontario, Toronto, Ontario, Canada; 4 Department of Public Health Sciences, Faculty of Medicine, University of Toronto, Ontario, Canada; 5 Department of Laboratory Medicine and Pathobiology, Faculty of Medicine, University of Toronto, Ontario, Canada

## Abstract

**Background:**

cMyc and p27 are key genes implicated in carcinogenesis. Whether polymorphisms in these genes affect breast cancer risk or prognosis is still unclear. In this study, we focus on a rare non-synonymous polymorphism in cMyc (N11S) and a common polymorphism in p27 (V109G) and determine their role in risk and prognosis using data collected from the Ontario Breast Cancer Family Registry.

**Methods:**

Risk factor data was collected at baseline on a large group of women (cases = 1,115 and population-based controls = 710) and clinical data (including treatment and follow-up) were collected prospectively by periodic review of medical records for a subset of cases (N = 967) for nearly a decade. A centralized pathology review was conducted. Unconditional logistic regression was used to determine the association of polymorphisms with breast cancer risk and the Cox proportional hazards model was used to determine their association with survival.

**Results:**

Our results suggest that while cMyc-N11S can be considered a putatively functional polymorphism located in the N-terminal domain, it is not associated with risk, tumor characteristics or survival. The p27-G109 allele was associated with a modest protective effect in adjusted analyses and higher T stage. We found no evidence to suggest that p27-V109G alone or in combination with cMyc-N11S was associated with survival. Age at onset and first-degree family history of breast or ovarian cancer did not significantly modify the association of these polymorphisms with breast cancer risk.

**Conclusion:**

Further work is recommended to understand the potential functional role of these specific non-synonymous amino acid changes and a larger, more comprehensive investigation of genetic variation in these genes (e.g., using a tagSNP approach) in combination with other relevant genes is needed as well as consideration for treatment effects when assessing their potential role in prognosis.

## Background

Several studies have implicated cMyc and p27 in breast cancer [1,2]. cMyc is amplified in 20–30% of breast tumors and amplification has been correlated with pre-menopausal status, specific tumor features (i.e., high tumor grade, lymph-node metastases, large tumor size and negative progesterone receptor status) and worse prognosis [1]. Loss of p27 expression is also a common event in breast cancers, and has been strongly associated with high tumor grade and poor prognosis [2].

Whether genetic variation in these two genes affects cancer risk or prognosis is not yet known. To our knowledge, only seven studies have examined polymorphisms in cMyc and p27 and all except one, which looked at haplotypes in p27, have focused on either cMyc-N11S or p27-V109G [3-9]. cMyc-N11S was recently reported by Wirtenberger et al. (2005) to be associated with non-BRCA familial breast cancer [3], but has not been investigated as a potential prognostic factor. A few studies have investigated the association of the p27-V109G polymorphism with cancer risk and progression, but results have been inconsistent [4-7]. A previous publication by our group reported no association for p27-V109G and breast cancer risk in a smaller sample of breast cancer cases (N = 398) and controls (N = 372) [8], but were unable to explore the association with tumor characteristics and survival. Ma et al. (2006) also showed no association of this polymorphism with breast cancer risk among Chinese women [9]. Another breast cancer study observed that the p27-V109G polymorphism was correlated with nodal involvement, but not with p27 tumor expression [4]. In univariate analysis among the node-negative group, V109G was significantly associated with shorter disease-free survival [4].

In this study, we explore whether these non-synonymous single nucleotide polymorphisms (nsSNPs) in cMyc (N11S) and p27 (V109G) are important risk and prognostic factors in breast cancer using a large, population-based cohort of incident breast cancer with systematically collected clinical data from the Ontario Familial Breast Cancer Registry (OFBCR).

## Methods

### Study design and subjects

Our study sample consisted of incident histo-pathologically confirmed cases of primary breast cancer from the population-based OFBCR [10,11]. Recruitment of cases and controls has been described previously [12-14]. In brief, all cases were identified from the Ontario Cancer Registry which registers >97% of all cases in the province. All women aged 20–54 years who met the OFBCR definition for high genetic risk (family history of specific cancers particularly breast and ovarian, early onset disease, Ashkenazi ethnicity or a diagnosis of multiple breast cancer) were asked to participate by completing risk factor questionnaires and providing a blood sample. A 25% random sample of individuals in this age category who did not meet the OFBCR definition, 35% of those aged 55–69 at high risk and 8.75% aged 55–69 at low risk were also asked to participate. This multi-step sampling scheme enriched the population for genetically predisposed individuals, which was an objective of the Ontario Familial Breast Cancer Registry [11]. Response rates were as follows: consent to contact patients was 92%, response to initial family history questionnaire was 65%, response to risk factor questionnaires was 73% of all eligible, and donation of a blood sample was 63% of all eligible. Less than 2% died before initial contact.

To conduct case-control studies, the OFBCR also collected unrelated, unaffected population controls (N = 710). They were recruited by calling randomly selected residential telephone numbers throughout the same geographical region. Eligible controls were women with no history of breast cancer and characteristics of the control population have been shown to be representative of the target population [14]. Approximately, 65% of identified eligible women returned questionnaires, and 63% of these donated a blood specimen.

For the prognostic study, those patients who provided a blood sample, had no prior malignancy (except for breast carcinoma-in-situ, non-melanoma skin cancer or cervix carcinoma-in-situ) and consented to retrieval of medical records were followed prospectively for clinical outcomes. The study methodology has been reported elsewhere [15]. In brief, clinical factors including stage, surgical treatment, radiation therapy, chemotherapy, and hormonal therapy were extracted from patient medical records by registered nurses at each clinic using validated data collection forms. Tumor pathological factors including tumor size, grade, number of positive lymph nodes, histologic subtype, status of margins, lymphatic and blood vessel invasion and hormone receptor status (estrogen, progesterone) were extracted from pathology reports and also obtained from a review of histologic slides by study pathologists. Medical records were reviewed annually for the occurrences of new primary cancer, local-regional and distant recurrences, changes in treatment and vital status. Data were reviewed, verified and coded centrally. The expected relationships between established prognostic and predictive factors and clinical outcomes in breast cancer were observed [16]. Individuals who were missing clinical data or who were ineligible due to refusal to provide a blood sample or consent to access medical records did not differ on main tumor pathological variables, survival and other patient characteristics from those with complete clinical follow-up data [16]. In total, there are 967 individuals with clinical follow up data.

Approval for this study was obtained from the Research Ethics Board of Mount Sinai Hospital and the University of Toronto.

### Polymorphism selection and genotyping

We identified non-synonymous polymorphisms in cMyc and p27 that may be biologically relevant using the NCBI dbSNP database by considering whether they were deleterious changes using two bioinformatics tools: SIFT [17] and PolyPhen [18], which have been recently advocated to be useful tools in identifying potentially causal variants [19]. We identified cMyc-N11S (rs4645959 A>G dbSNP Build 123). We also decided to genotype p27-V109G (rs2066827 G>T dbSNP Build 125) which was not deemed to be deleterious using either tool, but has been previously studied in the published literature. The 5'nuclease Taqman assay was used and technical details will be provided upon request from the authors. Water control, internal controls and previously genotyped samples were included in each plate to ensure accuracy of genotyping. Positive and negative controls were used in each genotyping assay, and 10% of the samples were randomly selected to be duplicated with 100% concordance. In addition, a total of 236 cases were genotyped twice for both polymorphisms in two different laboratories (The Centre for Applied Genomics at the Hospital for Sick Children, Toronto and H.O.'s lab at Mount Sinai Hospital, Toronto) using the same technique, and concordance was 100%.

### Statistical analysis

Pearson's chi-squared test or Fisher's exact test was used to determine the association between genotypes and patient/tumor characteristics. Variables were defined according to standard convention in order to facilitate comparison with other published studies. Age was dichotomized at 50 years of age to represent the approximate age of menopause for stratified analysis. An individual was considered to have a family history of cancer if she had a first-degree female relative with breast or ovarian cancer at the time of diagnosis (or date of entry for controls) since first-degree family history has been shown to be valid and reproducible by self-report for breast cancer [20]. Stage was defined according to the American Joint Committee on Cancer Staging System (1988) and T stage was categorized as low (pT1, <2 cm) or high (pT2, pT3, pT4 or >= 2 cm), which is prognostically relevant in breast cancer. Nodal status was categorized as no regional lymph nodes affected (pN0) or at least one nodal metastasis. Histopathological grade was defined according to the Scarff, Bloom and Richardson definition (I: well differentiated, II: moderately differentiated and III: undifferentiated). Estrogen and progesterone receptor status (ER or PgR) were classified as negative, equivocal or positive; equivocal tumors were combined with positive tumors.

Genotype frequencies among the controls were tested for Hardy-Weinberg equilibrium (HWE) using Pearson's chi-square test with 1 *df*. We report association results for a co-dominant model unless there were few variant homozygotes. The associations between SNPs and breast cancer risk were estimated as odds ratios (OR) and 95% confidence intervals (95% CI) by unconditional logistic regression adjusting for age (years) and ethnicity (White, Other). We stratified by age and family history to compare risk estimates in each category. Tests for interaction by inclusion of the corresponding product terms in logistic models were non-significant (data not shown).

Contingency table analyses were used to examine the associations between selected tumor characteristics and genotypes among cases with complete clinical follow-up. The primary clinical outcomes were time to distant recurrence and death. Survival time was calculated from date of surgery to these endpoints, censoring at the date of last contact or date of non-breast primaries. The Cox proportional hazard model was used to evaluate the crude and covariate-adjusted associations of factors with survival. The final multivariable model included established prognostic factors regardless of level of significance or confounding. Family history and ethnicity were also included because of their potential association with genotype. Graphical evaluation by Schoenfeld's residual plot indicated that the proportional hazard assumption of the Cox model could not be rejected for any of the covariates. Stratified analyses by age or family history were not conducted due to limited sample size.

To account for the sampling design in this study we also conducted a weighted analysis using the inverse of the sampling fractions as weights, and found no material differences. Therefore, we report results only from the unweighted analysis. Furthermore, our associations remain unchanged if limited to non-Ashkenazi or non-BRCA cases (data not shown).

For analyses of combined cMyc and p27 alleles we report all possible combinations using the most common genotypes as the reference category in order to obtain stable risk estimates, and avoid testing all possible combinations.

A priori power calculations using Quanto [21] showed that our study (1,115 cases and 710 controls) had 75% and 99% statistical power to detect an odds ratio of 1.5 for polymorphisms with an allele frequency of 0.05 (approximate for cMyc-N11S) and 0.25 (p27-V109G) assuming the dominant model. In our prognostic study (967 cases), with a follow-up of 6 years, recruitment of 3 years and hypothesized failure rate of 15% we had 70% power for the cMyc polymorphism to detect a relative risk of 2.0 and 80% for the p27 polymorphisms to detect a relative risk of 1.6 [22].

Missing data for given variables were reported in tables if >10% and all tests were two-sided. We did not adjust for multiple testing since this study focused on a few a priori defined hypotheses.

## Results

The majority of breast cancer cases were White pre-menopausal women and slightly more than a third had a first-degree family history of breast or ovarian cancer (Table 1). There were 75 (7.8%) confirmed BRCA1 or 2 carriers among the cases. There were no significant differences between all cases and those with clinical follow-up data. Controls were more likely to have children compared to cases. Genotype frequencies for each polymorphism showed no deviation from HWE among controls.

**Table 1:** Characteristics of Breast Cancer Cases and Population Controls in the OFBCR

**Characteristic**	**All Cases (N = 1,115)**	**Cases with Clinical Data (N = 967)**	**All Controls (N = 710)**
Age^1^			
Mean ± SD	48.6 ± 9.0	48.9 ± 9.2	48.5 ± 9.1
Menopausal Status^2^			
Pre-	828 (75.0%)	710 (73.7%)	497 (70.2%)
Post-	276 (25.0%)	253 (26.3%)	211 (29.8%)
Ethnicity			
White	1062 (95.3%)	899 (93.0%)	653 (95.2%)
Non-White	53 (4.7%)	68 (7.0%)	33 (4.8%)
Parity			
Nulliparous	201 (19.5%)	204 (21.5%)	106 (15.0%)
1–2 child	620 (60.2%)	562 (59.2%)	434 (61.2%)
> 3 children	209 (20.3%)	183 (19.3%)	168 (23.8%)
First-degree Family History^3^			
No	731 (65.6%)	618 (63.9%)	641 (90.3%)
Yes	384 (34.4%)	349 (36.1%)	69 (9.7%)
OFBCR Defined Genetic Risk Case^4^			
No	311 (27.9%)	281 (29.1%)	NA
Yes	804 (72.1%)	686 (70.9%)	
Histology			
Infiltrating Ductal No Special Type	--	824 (90.2%)	NA
Lobular		65 (7.1%)	
Other Special Type^5^		25 (2.7%)	
T Stage			
pT1 (<2 cm)	--	607 (64.0%)	NA
pT2 (2–5 cm)		281 (29.6%)	
pT3/pT4 (>5 cm)		34 (3.6%)	
pTx (not accessible)		26 (2.7%)	
# Positive Lymph Nodes			
None	--	538 (56.6%)	NA
1–3		243 (25.6%)	
≥4		113 (11.9%)	
Nx (not accessible)		56 (5.9%)	
Grade			
I (well differentiated)	--	194 (21.5%)	NA
II (moderately differentiated)		351 (38.8%)	
III (poorly differentiated)		359 (39.7%)	
Lymphatic Vessel Invasion			
Negative	--	582 (66.1%)	NA
Positive		299 (33.9%)	
Estrogen Receptor Status			
Negative	--	230 (24.5%)	NA
Equivocal		49 (5.2%)	
Positive		659 (70.3%)	
Progesterone Receptor Status			
Negative	--	259 (27.8%)	NA
Equivocal		55 (5.9%)	
Positive		618 (66.3%)	

^1 ^age at diagnosis for cases and age at interview for controls; ^2 ^peri-menopausal women are grouped with pre-menopausal women; ^3 ^self-reported cancer histories of breast or ovarian cancer; ^4 ^criteria used by the OFBCR to oversample more informative cases in order to enrich registry for genetically predisposed individuals (see methods); ^5 ^includes medullary, tubular, cribriform, micropapillary, mucinous, metaplastic; NA, not applicable

cMyc-N11S genotypes were not associated overall with breast cancer risk (Table 2). The p27-G109 allele was associated with a significant, but modest protective effect in adjusted analyses [GT vs. TT: OR, 0.70 (0.52–0.93) and GG vs. TT: OR, 0.83 (0.42–1.65)]. Age at onset (under or over 50 years) and first-degree family history of breast or ovarian cancer did not significantly modify the association between these polymorphisms and breast cancer risk (data not shown). The combined effect of the two polymorphisms, using the most common genotype as the reference, did not show any relationship with risk, and a test for interaction between cMyc and p27 was non-significant. Estimates of risk were similar in the entire sample as when restricted to Caucasians.

**Table 2:** Association of Polymorphisms cMyc-N11S and p27-V109G with Breast Cancer Risk

	**Total**				**White**			
	
	**Cases (N = 1,115)**	**Controls (N = 710)**	**Unadjusted OR (95% CI)**	**Age and race-adjusted OR (95% CI)**	**Cases (N = 1,079)**	**Controls (N = 677)**	**Unadjusted OR (95% CI)**	**Age-adjusted OR (95% CI)**
**cMyc-N11S (A>G)**								
AA	1011 (91.9%)	653 (91.8%)	1.00	1.00	976 (91.7%)	621 (91.7%)	1.00	1.00
AG/GG	89 (8.1%)	57 (8.0%)	1.00 (0.71–1.43)	1.15 (0.73–1.82)	88 (8.4%)	56 (8.3%)	1.00 (0.71–1.42)	1.18 (0.75–1.87)
**p27-V109G (T>G)**								
TT	668 (61.5%)	405 (59.8%)	1.00	1.00	644 (61.1%)	393 (59.7%)	1.00	1.00
TG	366 (33.7%)	243 (35.9%)	0.91 (0.75–1.12)	0.70 (0.52–0.93)	359 (34.1%)	238 (36.2%)	0.92 (0.75–1.13)	0.71 (0.53–0.94)
GG	53 (4.9%)	29 (4.3%)	1.11 (0.69–1.77)	0.83 (0.42–1.65)	51 (4.8%)	27 (4.1%)	1.15 (0.71–1.87)	0.84 (0.42–1.67)
**Combined**								
cMyc AA + p27 TT	611 (56.4%)	369 (54.5%)	1.00	1.00	588 (56.0%)	357 (54.3%)	1.00	1.00
cMyc AA + p27 TG/GG	386 (35.6%)	253 (37.4%)	0.92 (0.75–1.13)	0.92 (0.74–1.13)	377 (35.9%)	246 (37.4%)	0.93 (0.76–1.15)	0.70 (0.52–0.94)
cMyc AG/GG + p27 TT	55 (5.1%)	36 (5.3%)	0.92 (0.59–1.43)	0.96 (0.61–1.51)	54 (5.1%)	36 (5.5%)	0.91 (0.59–1.42)	0.98 (0.55–1.74)
cMyc AG/GG + p27 TG/GG	32 (3.0%)	19 (2.8%)	1.02 (0.57–1.82)	1.08 (0.59–1.98)	32 (3.0%)	19 (2.9%)	1.02 (0.57–1.83)	0.99 (0.45–2.19)

Table 3 shows the relationships between selected tumor characteristics with the polymorphisms. There were no differences by genotypes for cMyc-N11S. The p27-G109 allele was associated with high T stage (p = 0.01) and possibly with nodal involvement (p = 0.07). Similar results as shown for ER status were observed if the data were analyzed for PgR or combined ER and PgR status (data not shown).

**Table 3:** Association of Polymorphisms cMyc-N11S and p27-V109G with Tumor Characteristics (N = 967)

	**T Stage^1^**		**Nodal Involvement**		**Grade**		**Estrogen Receptor^2^**	
	**Low (N = 607)**	**High (N = 315)**	**None (N = 538)**	**>= 1 (N = 356)**	**I (N = 196)**	**II&III (N = 710)**	**Negative (N = 279)**	**Positive (N = 659)**

**cMyc-N11S (A>G)**								
AA	553(92.6%)	279(89.7%)	490(91.9%)	315(91.0%)	174(90.2%)	642(92.2%)	249(90.2%)	600(92.6%)
AG/GG	44 (7.4%)	32 (10.3%)	43 (8.1%)	31 (9.0%)	19 (9.8%)	54 (7.8%)	27 (9.8%)	48 (7.4%)
*p-value*	0.13		0.64		0.35		0.23	
**p27-V109G (T>G)**								
TT	371(62.8%)	177(56.9%)	334(63.1%)	197(57.4%)	116(60.7%)	423(61.1%)	166(59.9%)	399(62.3%)
TG	198(33.5%)	109(35.1%)	174(32.9%)	122(35.6%)	66 (34.6%)	234(33.8%)	99 (35.7%)	206(32.2%)
GG	22 (3.7%)	25 (8.0%)	21 (4.0%)	24 (7.0%)	9 (4.7%)	35 (5.1%)	12 (4.3%)	35 (5.5%)
*p-value*	0.01		0.07		0.97		0.50	

^1 ^Low (pT1-2 or < 2 cm) and High (pT3-pT4 or > 2 cm), pTx excluded; ^2 ^equivocal results grouped with positives.

There was no association between any of the polymorphisms or their combined alleles and either distant-recurrence free survival or overall survival (Table 4). Tests for interaction between cMyc and p27 allele in these models were non-significant. There was no association between polymorphisms and survival within treatment groups: radiation, chemotherapy or hormonal therapy (data not shown). There were also no material differences if we examined these associations stratified by nodal status or ER/PgR status (data not shown).

**Table 4:** Association of Polymorphisms cMyc-N11S and p27-V109G with Breast Cancer Survival

	**Distant Recurrence Free Survival**			**Overall Survival**		
	
	**N_R_/N**	**unadjusted HR (95% CI)**	**adjusted^1 ^HR (95% CI)**	**N_D_/N**	**unadjusted HR (95% CI)**	**adjusted^1 ^HR (95% CI)**
**cMyc-N11S (A>G)**						
AA	108/861	1.00	1.00	77/876	1.00	1.00
AG/GG	5/74	0.54 (0.22–1.32)	0.50 (0.20–1.23)	5/76	0.77 (0.31–1.91)	0.63 (0.23–1.75)
*p-value*		0.17	0.13		0.58	0.38
**p27-V109G (T>G)**						
TT	67/570	1.00	1.00	47/579	1.00	1.00
TG/GG	46/367	1.05 (0.72–1.53)	1.00 (0.68–1.48)	35/375	1.11 (0.72–1.72)	0.97 (0.61–1.55)
*p-value*		0.79	0.99		0.65	0.90
**Combined**						
cMyc AA + p27 TT	63/522	1.00	1.00	43/529	1.00	1.00
cMyc AA + p27 TG/GG	45/330	3.34 (0.46–24.1)	3.83 (0.53–27.7)	34/338	2.23 (0.31–16.2)	2.57 (0.35–18.8)
cMyc AG/GG + p27 TT	4/45	3.70 (0.51–26.8)	3.71 (0.51–27.0)	4/47	2.64 (0.36–19.3)	2.45 (0.33–18.0)
cMyc AG/GG + p27 TG/GG	1/28	2.40 (0.27–21.5)	2.37 (0.27–21.3)	1/28	2.37 (0.26–21.2)	2.35 (0.26–21.1)
*p-value*		0.51	0.47		0.73	0.83

N number at risk; N_R _number of distant recurrences; N_D _number of deaths; ^1 ^adjusted for age, nodal involvement, grade, ethnicity, treatment and T stage

## Discussion and conclusion

There has been great interest in understanding the role of cMyc and p27 amplification/expression in breast cancer risk and prognosis, but surprisingly little on the role of polymorphisms. Our study fills this gap by presenting findings on the role of two specific polymorphisms in these genes. Our data suggests that the p27-G109 allele may confer a protective effect against breast cancer. This observation needs to be confirmed by other breast cancer studies, since there is disagreement in the published literature about its potential role. A previous publication by our group showed no association with a smaller sample size [8], as did a study by Ma et al. (2006) among Chinese women (cases = 368, controls = 467) [9]. Furthermore, one case-control study of prostate cancer (cases = 92, controls = 106) found a positive association with this polymorphism especially in cases under 66 at the time of diagnosis [7], while a family-based study of hereditary prostate cancer (N = 188 families) that resequenced p27 did not confirm this association, but identified the -32T polymorphism in the promoter site as a risk factor especially among cases diagnosed under age 65 [6]. Another study of oral squamous cell carcinoma did not find an association between p27-V109G and risk (cases = 713, controls = 1,224), but did show an association with overall tumor stage [5]. In this study, we also show that p27-V109G is associated with T stage and possibly nodal status, which was previously reported by Schondorf et al. [4]. Furthermore, our data do not suggest that the p27-G109 allele is associated with breast cancer survival, which confirms the overall null association with breast cancer survival as reported previously [4]. The latter study; however, did show a significant association with distant recurrence free survival among the node-negative tumors (N = 46). We did not confirm this finding with a larger, but still limited sample of node-negative cases.

Previous studies have shown that reduced p27 expression correlates with poor clinical outcomes, invasiveness, poor prognosis, high tumor grade and progression in breast cancer [2]. The missense V109G change may alter the interaction between p27 and its negative regulator p38^jab1 ^because it is located in the interaction surface [23]. Since p27 is rarely mutated and decreased protein levels are found in tumors, it can be hypothesized that this decrease may be the result of changes in degradation of p27. Therefore, the V109 allele may alter p27 affinity for p38^jab1 ^and thereby modify p27 degradation. However, since data from a homology-based bioinformatic tool suggests that this amino acid substitution is not deleterious [24], this is a hypothesis that needs to be confirmed in functional studies.

Our results do not confirm the recent findings by Wirtenberger et al. (2005) [3], who found that an increased risk for breast cancer associated with the S11 allele (OR = 1.54, 95% CI, 1.05–2.26) and a stronger effect among women > 50 years (OR = 2.24, 95% CI, 1.20–4.21) [3]. This lack of replication could be due to differences in study design including the selection of the study population. Wirtenberger et al. focused on non-BRCA1/2 familial cases selected from two countries (n = 349 Polish; n = 356 German) and non-BRCA1/2 healthy controls (n = 441 Polish; n = 655 German) collected from clinics from 1997–2003. The current investigation is this population-based study of incident breast cancer cases and unrelated controls in Canada. To be more directly comparable, we also excluded the 75 BRCA1/2 cases, but the results remained unchanged. No other study has been published on polymorphisms in cMyc and cancer risk.

Our study also showed that the cMyc-N11S polymorphism was not related to any patient or tumor characteristic or prognosis. No other study has been published on this topic. In general, the role of cMyc in breast cancer prognosis is unclear. cMyc can direct cells to either proliferation, differentiation or apoptosis [25,26]. However, cMyc engenders different proteins that may have different, and even opposite functions depending on the context, and we do not presently know the function of the highly conserved amino acid at position 11. A recent analysis of the N-terminal domain of cMyc suggests that mutants missing amino acids 1–100 are less able to induce apoptosis and growth, and less able to repress c-myc and gadd45α than the wild-type [27]. Furthermore, in this study mutants missing Myc Box 1 (amino acids 45–63 and 55–92) did not explain these results, as they were no different from the wild-type, suggesting that amino acids 1–45 are critical for these functions. But the effect of substitution with serine (S) residue at position 11 compared to asparagine (N) is unknown and merits further functional analysis, but may affect cMyc's ability to direct apoptosis.

We believe this study represents an important contribution to the published literature. We investigated two polymorphisms that can be considered to be strongly biologically relevant in breast cancer. However, our study is limited in statistical power for the survival analysis as noted by the wider confidence intervals, and we cannot exclude the possibility of very small effects of these polymorphisms (OR<1.5). Nevertheless, this study still represents, to our knowledge, the largest study of cMyc-N11S in cancer risk and the only study examining prognostic effects in breast cancer. This is the largest investigation of the p27-V109G polymorphism in cancer risk and prognosis. It is also important to note that this study is investigating only two nsSNPs and indirectly any SNPs in strong linkage disequilibrium. Over-sampling cases that are likely to be genetically predisposed may be considered a limitation in terms of the generalizability of our results, but we have shown that genetic risk is not associated with survival in this cohort, and therefore cannot be a source of confounding [16]. Furthermore, previous studies have shown minimal evidence of selection bias in this cohort [13,28]. As in all observational studies as opposed to clinical trials we do not have uniform treatment nor a standard evaluation of clinical outcomes, but our regular follow-up and high quality data collection are strengths of this study.

Our results do not support the hypothesis that these specific nsSNPs are strong factors influencing breast cancer risk or prognosis, although there is some suggested protective effect of the p27-G109 allele in risk. We have three recommendations for future studies: (i) a thorough functional analysis of the effect of these nsSNPs; (ii) focus on polymorphisms in coding or the promoter regions and the identification of those variants that correlate with intermediate phenotypes such as cMyc amplification and p27 over-expression, which are clinically relevant; and (iii) a comprehensive study of the association between genetic variation of these genes and breast cancer prognosis with consideration for the effect of treatment (i.e, p27 and herceptin [29]), which will necessitate the conduct of large, collaborative studies.

## Competing interests

The author(s) declare that they have no competing interests.

## Authors' contributions

JCF participated in the defining the study objectives and design, performing the statistical analysis and writing the manuscript. JAK assisted in outlining the study objectives and design and manuscript writing. SC and SS assisted in the genotyping and manuscript writing with respect to the functional and bioinformatic aspects. LB assisted with the statistical analysis. UVO assisted in the genotyping of the p27 polymorphism. PJG assisted with study design of the prognostic study. JRM assisted with defining the study objectives and design and manuscript writing. ILA assisted with the study objectives and design and manuscript writing. HO assisted with the genotyping and manuscript writing. All authors read and approved the final manuscript.

## Pre-publication history

The pre-publication history for this paper can be accessed here:


